# Immune subtyping for pancreatic cancer with implication in clinical outcomes and improving immunotherapy

**DOI:** 10.1186/s12935-021-01824-z

**Published:** 2021-02-26

**Authors:** Jingkai Liu, Qiaofei Liu, Xiang Zhang, Ming Cui, Tong Li, Yalu Zhang, Quan Liao

**Affiliations:** Department of General Surgery, Peking Union Medical College Hospital, Peking Union Medical College, Chinese Academy of Medical Sciences, Beijing, 100730 China

**Keywords:** Pancreatic cancer, Heterogeneity, Immune cell, Immune checkpoints, Immunotherapy, Transglutaminase 2

## Abstract

**Background:**

Emerging evidence has shown that intra-tumor immune features are associated with response to immune checkpoint blockade (ICB) therapy. Accordingly, patient stratification is needed for identifying target patients and designing strategies to improve the efficacy of ICB therapy. We aimed to depict the specific immune features of patients with pancreatic cancer and explore the implication of immune diversity in prognostic prediction and individualized immunotherapy.

**Methods:**

From transcriptional profiles of 383 tumor samples in TCGA, ICGC, and GEO database, robust immune subtypes which had different response immunotherapy, including ICB therapy, were identified by consensus clustering with five gene modules. DEGs analysis and tumor microarray were used to screen and demonstrate potential targets for improving ICB therapy.

**Results:**

Three subtypes of pancreatic cancer, namely cluster 1–3 (C1–C3), characterized with distinct immune features and prognosis, were generated. Of that, subtype C1 was an immune-cold type in lack of immune regulators, subtype C2, with an immunosuppression-dominated phenotype characterized by robust TGFβ signaling and stromal reaction, showed the worst prognosis, subtype C3 was an immune-hot type, with massive immune cell infiltration and in abundance of immune regulators. The disparity of immune features uncovered the discrepant applicability of anti-PD-1/PD-L1 therapy and potential sensitivity to other alternative immunotherapy for each subtype. Patients in C3 were more suitable for anti-PD-1/PD-L1 therapy, while patients in the other two clusters may need combined strategies targeted on other immune checkpoints or oncogenic pathways. A promising target for improving anti-PD-1/PD-L1 treatment, TGM2, was screened out and its role in the regulation of PD-L1 was investigated for the first time.

**Conclusion:**

Collectively, immune features of pancreatic cancer contribute to distinct immunosuppressive mechanisms that are responsible for individualized immunotherapy. Despite pancreatic cancer being considered as a poor immunogenic cancer type, the derived immune subtypes may have implications in tailored designing of immunotherapy for the patients. TGM2 has potential synergistic roles with ICB therapy.

## Introduction

Pancreatic ductal adenocarcinoma (PDAC) has been striking a heavy burden on human health by increasing worldwide incidence and less than 9% survival rate [1]. Advances in chemotherapy regimens over the last two decades have only modestly prolonged the overall survival of the patients [2]. More effective treatments are still needed for PDAC patients. A promising ICB therapy, programmed cell death protein 1 (PD-1)/programmed death 1 ligand 1(PD-L1) antibodies, has yielded significant clinical efficacy in some tumor types [3]. However, low response rate and limited patients benefited from single-agent ICB were observed in PDAC, which can be attributed to the low immunogenicity and diverse immunosuppression mechanisms [4]. To overcome the drug resistance, combination with other ICB targets or other therapeutic modalities has been regarded as a hopeful solution [5]. While the precondition for making an appropriate combination treatment strategy is a reasonable method for patient stratification based on similar characteristics of the immune response.

Over the last decade, substantial progression in molecular subtyping for PDAC has facilitated the understanding of molecular pathogenesis and provided clues for advanced therapy designing [6]. However, to date, there is still lacking an immune feature-based molecular subtyping for better understanding the heterogeneity of immune response and reasons for the inefficiency of ICB therapy.

A recent pan-cancer study revealed a widely suitable immune-subtyping method based on five immune signatures which provided a potential roadmap for PDAC [7]. Herein three distinct immune subtypes of PDAC were presented based on these five immune signature modules. Each subtype showed distinct immune cell composition and expression patterns of immunomodulators, which provided a reasonable explanation for their survival discrepancy and inefficacy of single-agent ICB therapy. Furthermore, we screened out a potential immunosuppression-related gene, transglutaminase 2 (TGM2). TGM2 is a multifunctional enzyme that, in addition to catalyzing protein crosslinking, can also serve in the regulation of signaling pathways by constitutively activating the key modulators, such as p53, Akt, and NF-κB [8–13]. The aberrant expression of TGM2 has been demonstrated to be linked with a series of aggressive phenotypes of tumor cells, such as tumor growth, metastasis, epithelial-mesenchymal transition, and cancer stem cell property [12, 14]. In vivo, based on orthotopic xenograft mouse models, downregulating the expression of TGM2 by siRNA or shRNA could restrain the tumor growth and improve the treatment effect of Gemcitabine in PDAC [9, 15, 16]. At present, whether or how TGM2 is involved in the immune regulation of PDAC remains unknown. Therefore, we explored the association of TGM2 with the immune microenvironment and investigated its potentially synergistic roles with ICB therapy.

Taken together, these findings provide a conceptional framework to understand the immune response diversity in the tumor microenvironment of PDAC, implicate PDAC patient stratification, and design combination therapeutic strategies based on ICB therapy.

## Materials and methods

### Transcriptional data resources and preprocessing

**Fig. 1:**
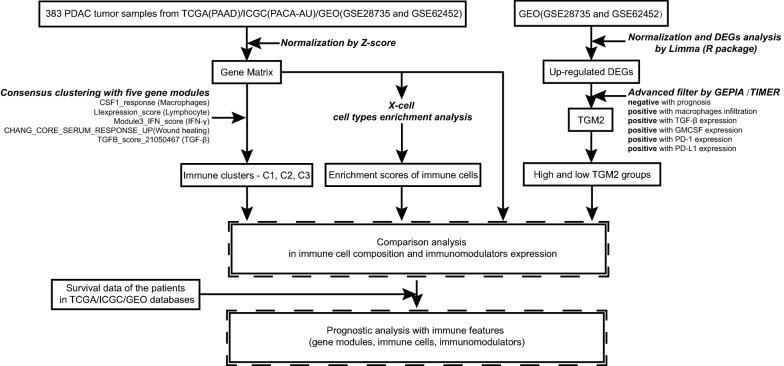
Flowchart of data collection and analysis in present study. Transcriptional profiles were collected from TCGA (PAAD)/ICGC (PACA-AU)/GEO (GSE28735 and GSE62452). Three immune clusters were derived by consensus clustering with five gene modules. The enrichment scores of cell types were calculated by X-cell. Then, comparison analysis in immune cell composition and immunomodulators expression were performed in groups and whole cohort. Combined with survival data of the patients TCGA/ICGC/GEO cohort, the prognostic analysis with immune features (gene modules, immune cells, and immunomodulators) were performed. To find promising targets for anti-PD-1/PD-L1 treatment, DEGs analysis and correlation analysis were performed in GEO (GSE28735 and GSE62452) database. The results led to gene TGM2, an oncogenic target for PDAC. The comparison analysis and prognostic analysis were performed in high-/low- TGM2 groups

Human pancreatic cancer transcriptional profiles were downloaded from public databases, including The Cancer Genome Atlas (TCGA, https://portal.gdc.cancer.gov/), International Cancer Genome Consortium (ICGC, https://icgc.org/) and Gene Expression Omnibus (GEO, https://www.ncbi.nlm.nih.gov/geo/). Read count data from TCGA (PAAD) and ICGC (PACA-AU) were normalized in the TMM method by edgeR (R package). Then TPM value was calculated respectively and the batch effect was eliminated by Combat. There were 182 TCGA data samples, of which 4 para-cancer samples and 1 non-primary sample were excluded, and finally, a total of 177 tumor samples were collected. GSE28735 and GSE62452, which are two datasets downloaded from the GEO database with the entire clinical following information, were integrated. The batch effect was eliminated by Combat. The combined datasets of GSE28735 and GSE62452 contained a total of 220 samples, including 114 tumor samples and 106 para-cancer samples. Together with 92 tumor samples from ICGC and 177 tumor samples from TCGA, 383 tumor samples were taken into analysis. To accomplish a combined analysis of tumor statistics, RNA-Seq and gene array transcriptional data were normalized in the manner of z-score. The flow chart of data processing was shown in Fig. 1.

### Patient cohort

The use of clinical samples and clinical information for this study was approved by the institutional review board of Peking Union Medical Hospital (NO. JS-987). Totally, 97 pairs of tumor and para-tumor normal samples of patients diagnosed with PDAC were collected as the description of our previous study [17]. All of the cases were pathologically confirmed to be PDAC, and R0 radical resection was achieved and at least three cycles of adjuvant chemotherapy were performed for each patient. Histological grading was made according to the 8th edition of the TNM system established by the American Joint Committee on Cancer (AJCC) [18]. None of the patients undergone neoadjuvant chemotherapy.

### Immunohistochemistry (IHC) and digital analysis

Immunohistochemistry staining was implemented in accordance with the previous study [17]. The microarray chip was stained with anti-TGM2 (15100-1-AP, Proteintech, 1:200). Quant Center identified and analyzed the areas of strong positive, moderately positive, weak positive, and negative pixels, as well as the percentage of positive pixels, and finally conducted an H-score.

### Cell culture

Human pancreatic cancer cell lines, PANC-1 and Mia PaCa-2, were maintained in DMEM medium supplemented with 10% fetal bovine serum and 1% penicillin and streptomycin and cultured in the incubator with 5% CO_2_ at 37 °C.

### Western blotting

Total protein was extracted using 2% SDS lysis buffer including protein phosphatase inhibitor (Applygen, Beijing) and heated for 10 min. Protein samples were separated by 8% (v/v) SDS-PAGE gels and transferred onto nitrocellulose membranes (Millipore, Ireland). After blocking in 5% (w/v) Albumin Bovine-V (BSA-V, Solarbio, China) for 1 h at room temperature, the protein bands were incubated with primary antibodies at a dilution ratio of 1:1000 overnight at 4 °C. The primary antibodies contain anti-GAPDH (FL-335; Santa Cruz Bio technology), anti-TGM2 (Proteintech), anti-PD-L1 (Proteintech), anti-STAT3 (CST) and anti-Phospho-STAT3 (Ser705, CST), anti-Akt (CST), and anti-Phospho-Akt (Ser473, CST), anti-P65 (Abcam) and anti-Phospho-P65 (Ser536, Abcam). The protein bands were incubated in HRP-conjugated secondary antibodies (Zsbio, China, 1:5000) at room temperature for 1 h.

### Transfection assay

Lentiviral particles for the TGM2 knockdown assay were purchased from Syngen Tech (Beijing, China). Lentiviral particles were added into PANC-1 cells and Mia PaCa-2 cells supplemented with 5 μg/ml polybrene. After 48 h of transfection, target cells were selected with 1 μg/ml of puromycin for two weeks. The TGM2 knockdown in cells was validated by Western blotting. The sequence of shTGM2 is acquired from a previous study and showed as below: 5′-AAGGGCGAACCACCTGAACAA-3′ [15].

### Statistical analysis

#### Identification of immune subtypes

Five immune signature sets, including CSF1_response (Macrophages), LIexpression_score (Lymphocyte), Module3_IFN_score (IFN-γ), TGFB_score_21050467 (TGF-β) and CHANG_CORE_SERUM_RESPONSE_UP (Wound healing) modules, were selected to run clustering analysis. They respectively represented the activation of macrophage/monocytes, overall lymphocyte infiltration, TGF-β response, IFN-γ response, and wound healing activity in tumor immune microenvironment. GSVA enrichment analysis was conducted and ssGSEA values were calculated. Unsupervised clustering with ssGSEA values of samples was conducted by McLust R package, and K values corresponding to maximized Bayesian Information criterion (BIC) were selected to obtain immune subtypes.

#### Depiction of molecular and cellular signatures in immune subtypes

To further understand the cellular and molecular characteristics of the immune microenvironment, we assessed the enrichment degree of 75 immunomodulatory genes and 64 immune cell types. The enrichment of immune cell types and immune-related genes were analyzed by xCel software and ImmuneCellAI (http://bioinfo.life.hust.edu.cn/ImmuCellAI) and R software. Kruskal–wallis test or Wilcox test were used to analyze the differences in enrichment scores of immune cells or immunomodulatory genes among different groups. Boxplot was drawn for the distribution of immune cells. The accuracy of immune cells in predicting survival was analyzed by C-index analysis.

#### Prognostic analysis

To evaluate the impact of immune signatures on patients’ survival, we performed Kaplan–Meier analysis, univariate and multivariate COX analysis. The results were presented as the mean ± standard deviation. C-index was used to analyze the accuracy of the gene sets model in predicting survival. *p* < 0.05 was considered statistically significant.

#### Identification of immune related gene target

Based on the gene expression profiles of GSE28735 and GSE62452, differentially expressed genes (DEGs) were collected by limma (R package) with filtration of *p-*value < 0.05 and Fold Change (FC) > 1. To identify target genes among the DEGs, further filtration was set as negative relation with prognosis, positive relation with the infiltration of macrophages and the expression of PD-L1, PD-1, TGF-β1 and GM-CSF and analyzed by the online tool of GEPIA (http://gepia.cancer-pku.cn/) and TIMER (https://cistrome.shinyapps.io/timer/). Set median expression value of target gene as cutoff and we divided the whole samples into high and low expression groups. The distribution of high and low groups in three immune subtypes then was described in a percentage and count manner. The prognostic analysis of the target gene was conducted by survival R package. The influence of immune modulator genes on patients’ survival in the above two high and low expression groups was also analyzed.

## Results

### Identification of immune subtypes in PDAC

PDAC harbored a highly heterogeneous tumor microenvironment. By performing clustering analysis with five immune signature gene sets, we classified three immune subtypes, namely C1–C3, in the 383 pancreatic tumor samples. The five immune signatures represented the activation of macrophage/monocytes (Macrophages), overall lymphocyte infiltration (Lymphocyte), TGF-β response (TGF-β), IFN-γ response (IFN-γ), and wound healing activity (Wound healing), respectively.

**Fig. 2:**
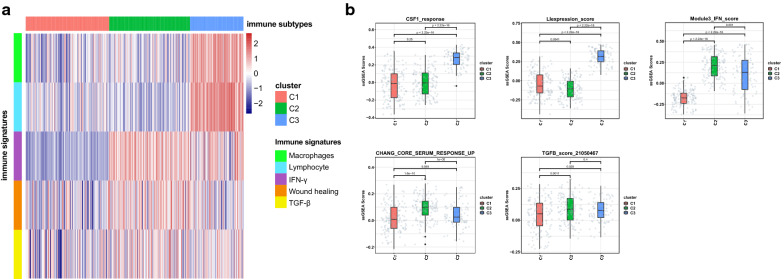
Immune subtypes of PDAC and immune signatures in TCGA and ICGC and GEO mix. **a** Immune subtypes of PDAC: column was for tumor samples and row was for five immune signatures. **b** Enrichment comparison of the five immune gene modules across three immune subtypes. Gene modules include CSF1_response (correspond to Macrophages), LIexpression_score (correspond to Lymphocyte), Module3_IFN_score (correspond to IFN-γ), CHANG_CORE_SERUM_RESPONSE_UP (correspond to Wound healing), and TGFB_score_21050467 (correspond to TGF-β)

Five immune signatures showed disparate enrichment patterns in three immune subtypes (Fig. 2a). Subtype C3 had the highest Lymphocyte and Macrophages as well as a favorable enrichment in IFN-γ, indicating an immune-hot phenotype (Fig. 2b). Subtype C1 had generally poor enrichment scores, especially the lowest enrichment in IFN-γ, TGF-β, and Wound healing, indicating an immune-cold phenotype (Fig. 2b). In comparison, subtype C2 had the highest TGF-β enrichment scores and the lowest Lymphocytes enrichment scores, indicating an immune-suppressive phenotype (Fig. 2b). Meanwhile, C2 also had the highest Wound healing enrichment scores indicating an active tissue remolding (Fig. 2b).

### Cellular composition and immunomodulators diversity of immune subtypes

**Fig. 3:**
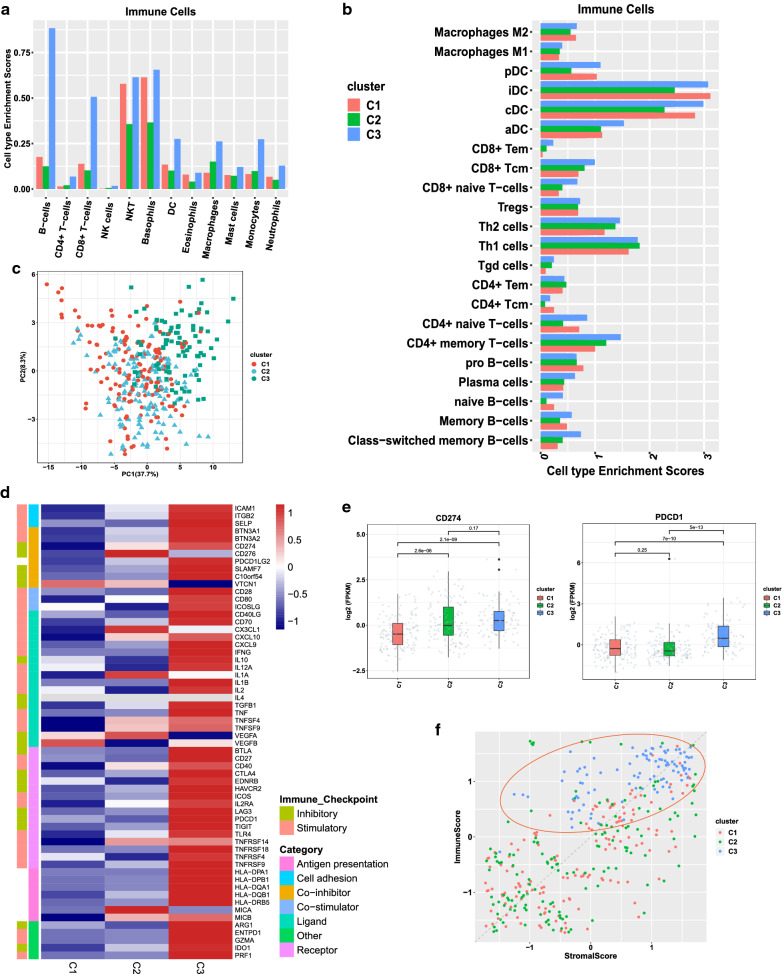
Immune cell composition and expression patterns of IMs in three subtypes. **a**, **b** Immune cell composition of three immune subtypes. **c** PCA analysis of IMs genes. **d** Expression patterns of IMs in three subtypes.** e** Enrichment disparity of CD274 and PDCD1 across the three subtypes. **f** Analysis of stromal and immune scores across the three subtypes: the distribution of samples in C3 was indicated by the red circle

Immune cell composition varied across three subtypes. Subtype C3, the immune-hot phenotype, had dominant cellular enrichment scores with the highest T lymphocytes (both CD8^+^ and CD4^+^), NKT cells, dendritic cells (DCs), macrophages, and B cells as well as granulocytes (Fig. 3a, b). Meanwhile, three subtypes displayed distinct expression patterns of immunomodulators (Fig. 3c). Compared with subtype C1 and C2, subtype C3 had higher CD27 and inducible synergistic co-stimulation molecules (ICOS) expression, indicating a favorable lymphocyte activation. In addition, higher IL2, IFN-γ, and TNF families (e.g. CD40) represents a favorable cellular immunity and anti-tumor ability of C3 (Fig. 3d). The expression pattern of inhibitory immune regulators varied from three subtypes. In addition to a higher TGF-β1, subtype C3 had higher immune checkpoint expression of CD274 (PD-L1, also as B7-H1), PDCD1 (PD-1), CTLA4, TIGIT, LAG3, HAVCR2 (TIM3) but lower expression of CD276 (B7-H3), and VTCN1 (B7-H4), compared with C1 and C2 (Fig. 3d, e).

Opposite enrichment patterns of immune-infiltrating cells were found between subtype C1 and C2. Subtype C1 had higher enrichment scores in T cells, NKT cells, DCs, and B cells but lower in macrophages, while C2 had higher scores in macrophages but lower scores in T cells, NKT cells, DCs, and B cells (Fig. 3a, b), indicating a stronger immunosuppressive microenvironment of subtype C2. On the other hand, both C1 and C2 showed a poor expression of stimulatory immunomodulators. As for the expression discrepancy of immune checkpoints, subtype C1 was mainly enriched in VTCN1, while C2 was dominated by CD274, CD276, and VTCN1 (Fig. 3d, e), which may have the clinical implication for further designing tailored immunotherapy strategy.

The stromal and immune scores of tumor samples were calculated by ESTIMATE software. Different from the discrete distribution of C1 and C2, subtype C3 was mostly enriched in the zones with high immune scores, which indicated a robust immune activity in C3 (Fig. 3f).

### Prognosis analysis of immune subtypes

**Fig. 4:**
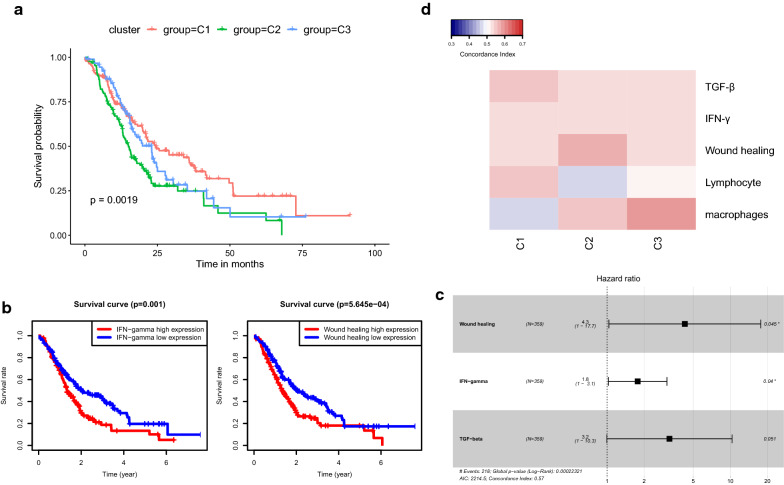
Prognostic impact of immune subtypes and immune signatures. **a** Overall survival of three immune subtypes. **b** Correlation of immune signatures with overall survival. The Left was for IFN-γ module. The Right was for Wound healing module. **c** Multivariate COX analysis of three immune signatures. Wound healing module (coef = 1.45, HR = 4.28, 95%CI 1.03–17.67, *p* = 0.045), IFN-γ module (coef = 0.57, HR = 1.77, 95%CI 1.03–3.06, *p* = 0.04), TGF-β module (coef = 1.16, HR = 3.21, 95%CI 0.99–10.34, *p* = 0.051). **d** Concordance index (CI) of five immune signature expression with the overall survival. Columns and rows represented immune subtypes and immune signatures respectively. Red indicated higher prediction accuracy while blue indicated lower prediction accuracy, along with the increasing expression of immune signature enrichment scores

The association between immune subtypes and patients’ survival was also analyzed. The immune-suppressive subtype C2 had the worst prognosis, while C1 and C3 had relatively favorable prognosis (C1:C2:C3, *p* = 0.0019, Fig. 4a; C1:C2, *p* = 0.00094; C1:C3, p = 0.24; C2:C3, *p* = 0.038, Additional file 1: Figure S1). The results of K-M analysis revealed that IFN-γ (*p* = 0.001) and Wound healing (*p* = 5.645e−04) modules were inversely correlated with patients’ overall survival (Fig. 4b). The univariate and multivariate cox analyses also showed the same results (Fig. 4c and Table 1). Furthermore, with concordance index (CI) analysis, we evaluated the validity of the five immune signatures in survival prediction. The results showed that IFN-γ, Wound healing and TGF-β modules had favorable prediction accuracy on patient survival among the three subtypes (Fig. 4d). Especially, Wound healing module in C2, Macrophages module in C3, TGF-β and Lymphocyte module in C1 seemed to be the best predictor for each subtype, respectively (Fig. 4d).

**Table 1:** Univariate and multivariate cox analyses of five immune signatures

Parameter	Univariate analysis			Multivariate analysis		
	HR	95%CI	P	HR	95%CI	P
Wound healing	9	2.38–34.01	*0.001*	4.28	1.03–17.67	*0.045*
Macrophages	1.29	0.67–2.44	0.438	–	–	–
Lymphocyte	1.06	0.60–1.86	0.846	–	–	–
IFN-γ	2.22	1.32–3.76	*0.003*	1.77	1.03–3.06	*0.04*
TGF-β	5.42	1.80–16.34	*0.003*	3.21	0.99–10.34	0.051

**Fig. 5:**
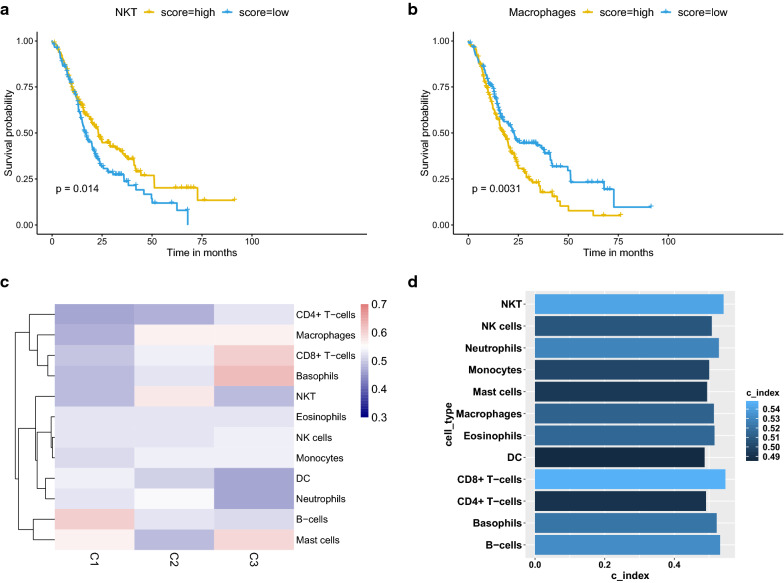
Prognostic impact of the immune cells. **a**, **b** Survival analysis of NKT cells (*p* = 0.014) and macrophages (*p* = 0.0031). **c** CI analysis with immune cells across the three subtypes: red indicated higher prediction accuracy while blue indicated lower prediction accuracy, along with the increasing expression of immune cells enrichment scores. **d** CI analysis with immune cells in the whole cohort: the lighter blue indicated greater prognostic impact while the darker blue indicated weaker prognostic impact

The prognostic analysis showed that NKT cells correlated with a favorable prognosis (*p* = 0.014) (Fig. 5a) while macrophages were associated with a poor prognosis (*p* = 0.0031) (Fig. 5b). The CI analysis showed that both NKT cells and macrophages could serve as favorable predictors for patients’ survival in C2 (Fig. 5c). Although other cell types showed no significant correlation with patients’ survival in our study (Additional file 1: Figure S2), immune cells especially CD8^+^ T cells, NKT cells, and B cells showed a greater impact on patients’ survival based on CI scores (Fig. 5d).

### TGM2 was involved in the immune-suppressive microenvironment in PDAC and high TGM2 expression predicted poorer survival

**Fig. 6:**
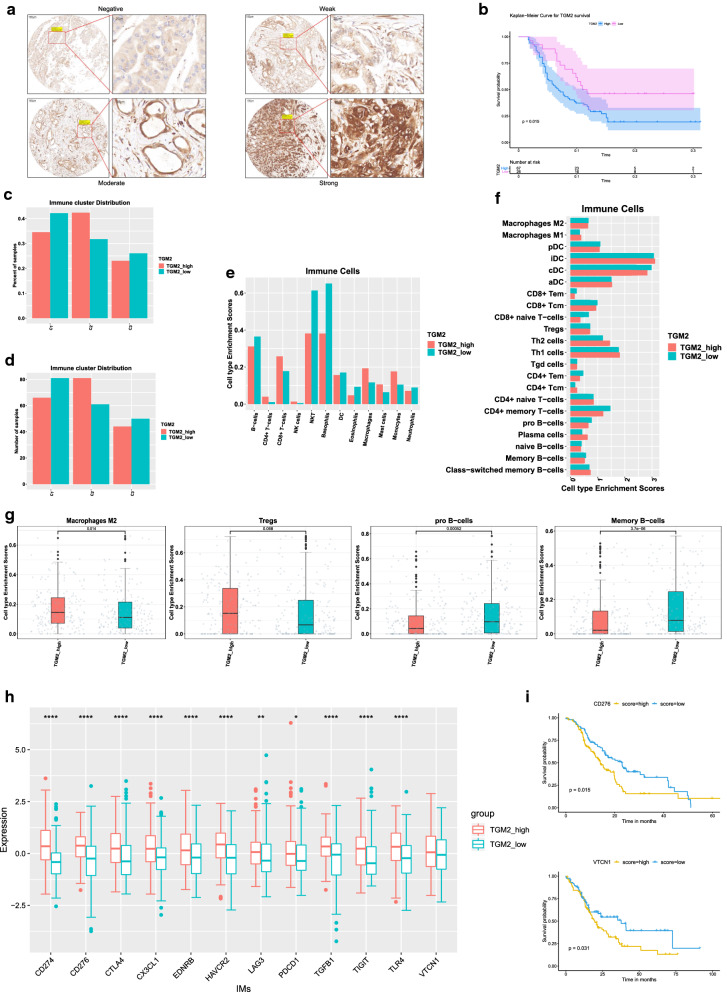
Relation between TGM2 and tumor immunosuppression in PDAC. **a** IHC analysis of TGM2 with our tissue microarray (*n* = 97). The scale bars were shown as indicated: 100 μm and 20 μm.** b** Survival analysis of TGM2 with tissue microarray data (*p* = 0.015). **c**, **d** Distribution of high- and low-TGM2 group across the three immune subtypes showed as percentage and number. **e, f** Immune cell composition of high- and low-TGM2 groups. **g** Enrichment comparison of M2 type macrophages (*p* = 0.014), Tregs (*p* = 0.068), pro-B cells (*p* = 0.00052) and memory B cells (*p* = 3.7e−06) between high- and low-TGM2 groups. **h** Comparison of IMs expression between high- and low-TGM2 groups: row is for the immunomodulators and column is for the gene expression value. (CD274, *p* = 1.0e−14; CD276, *p* = 1.1e−10; CTLA4, *p* = 5.6e−07; CX3CL1, *p* = 1.6e−06; EDNRB, *p* = 3.8e−06; HAVCR2, *p* = 4.3e−10; LAG3, *p* = 0.0046; PDCD1, *p* = 0.0121; TGFB1, *p* = 1.2e−12; TIGIT, *p* = 6.5e−06; TLR4, *p* = 3.8e−08; VTCN1, *p* = 0.0585). **i** Survival analysis of CD276 expression in high-TGM2 group (top) and VTCN1 expression in low-TGM2 group (bottom)

To find the underlying target for anti-PD-1/PD-L1 treatment, we performed DEGs analysis with the two GEO datasets (GSE62452 and GSE28735). Among all the up-regulated DEGs, following the set filters, the result led to the gene TGM2 (Additional file 1: Figure S3). With IHC analysis in our tissue microarray, we also verified that TGM2 was elevated in tumor tissue compared with adjacent normal tissue (Fig. 6a) and had a negative impact on prognosis (Fig. 6b). In addition, survival analysis within the TCGA-ICGC-GEO cohort verified the prognostic impact of TGM2 (Additional file 1: Figure S4).

Besides, the expression patterns of TGM2 across the three subtypes were different. Most patients comprising subtype C2 had high TGM2 expression, while subtype C1 and C3 were dominated by low TGM2 expression (Fig. 6c, d). Meanwhile, in the analysis of the immune cells composition between high- and low-TGM2 groups, we found that macrophages mostly enriched in the high-TGM2 group, while NKT cells mostly enriched in the low-TGM2 group (Fig. 6e). Furthermore, compared with the low-TGM2 group, the high-TGM2 group seemed to have higher M2 macrophages (*p* = 0.014) and Treg cells (*p* = 0.068) but lower pro-B cells (*p* = 0.00052) and memory B cells (*p* = 3.7e−6) (Fig. 6f, g). And high-TGM2 group harbored a higher expression pattern of inhibitory immunomodulators (CD274, CD276, CTLA4, EDNRB, HAVCR2, LAG3, PDCD1, TGFB1, TIGIT, and VTCN1) in the whole cohort (Fig. 6h). In the prognostic analysis with IMs between the two groups, higher CD276 was associated with worsen prognosis in the high-TGM2 group and higher VTCN1 was related to poorer overall survival in the low-TGM2 group (Fig. 6i). Together, these findings suggested that TGM2 may be involved in the immunosuppression in PDAC.

### TGM2 may regulate PD-L1 expression via STAT3/NF-κB signaling pathways in PDAC

**Fig. 7:**
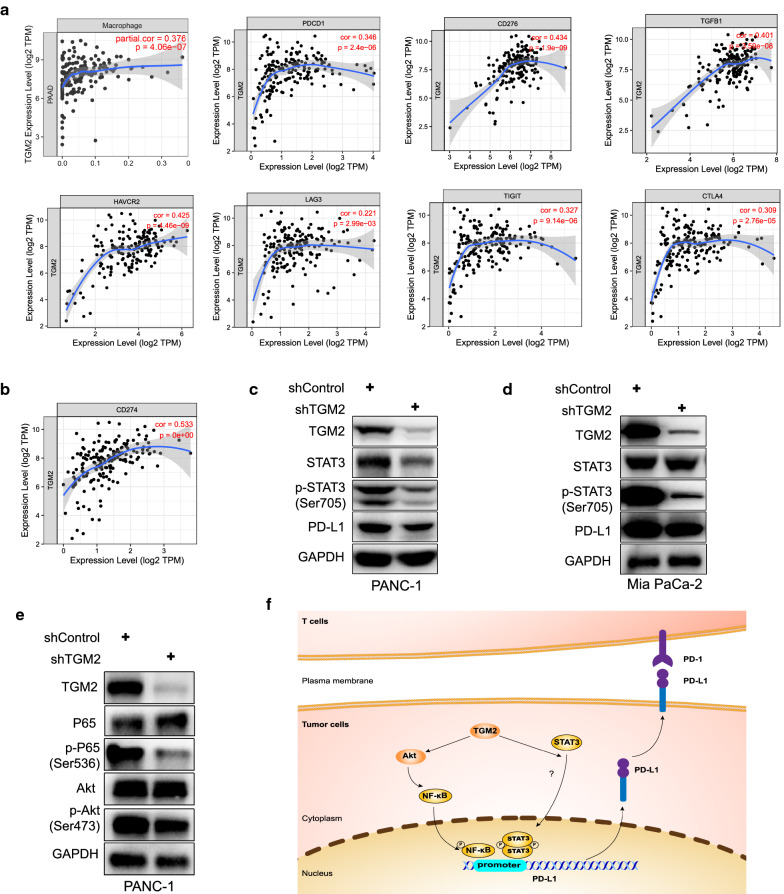
Proposed mechanism that TGM2 may regulate PD-L1 expression via STAT3/NF-κB signaling pathways in PDAC. **a** Correlation analysis of TGM2 and immunosuppressive factors with TIMER. **b** Correlation analysis of TGM2 and CD274 (PD-L1) by TIMER. **c**, **d** Western blot results showed that TGM2 knocking down resulted in a decreased expression of PD-L1 and p-STAT3 in PANC-1 and Mia PaCa-2 cells. **e** TGM2 knocking down in PANC-1 cells led to a decreased expression of p-Akt (Ser473) and p-P65 (Ser536) which was consistent with previous studies. **f** TGM2 may regulate PD-L1 via NF-κB/STAT3 signaling pathways: **a** TGM2 activated AKT pathway and then promotes the activation of downstream transcription factor NF-κB which has been reported to be able to directly bind with the promoter of PD-L1 and stimulate its transcription. **b** TGM2 may promote the phosphorylation of STAT3 despite the underlying pathways remains unclear, and then p-STAT3 binds to the promoter of PD-L1 and stimulate its transcription

To further explore the potential roles of TGM2 in immunosuppression, we analyzed the relation between TGM2 and the suppressive factors (Fig. 7a). The relative coefficient between TGM2 and PD-L1 is the most robust (Fig. 7b). We verified the correlation between TGM2 and PD-L1 in human PDAC cell lines (PANC-1 and Mia PaCa-2)*.* After TGM2 knocking-down in PANC-1, we found a decreased expression level of PD-L1 (Fig. 7c). In the cell line of Mia PaCa-2, we observed the same expression variation (Fig. 7d). STAT3 and NF-κB are two important transcriptional factors in tumor evolution and are reported to be capable to regulate the expression of PD-L1 [19, 20]. We knocked down TGM2 in PANC-1 and Mia PaCa-2 cells, a decreasing level of p-STAT3 was observed (Fig. 7c, d). The previous study demonstrated that TGM2 regulated the activation of NF-κB by modulating phosphorylation of AKT [10]. In our study, we proved that TGM2 knocking down resulted in a decrease of p-Akt (Ser473) and p-P65 (Ser536) (Fig. 7e).

## Discussion

ICB has become a promising immunotherapeutic modality for several refractory carcinomas, however, its roles in PDAC are limited. The challenges majored in the identification of target patients and finding effective combination therapeutic targets to amplify its clinical efficacy. In this study, we utilized published immune signature gene sets to depict the distinct immune features of PDAC and three immune subtypes are identified (the sample details of immune subtypes are shown in the Additional file 1). The prognostic impact of immune gene sets, immune cell composition, and immunomodulators are evaluated. The diversity of immune response reflects the inner cause of the prognostic discrepancy and immunotherapy failure in PDAC. Meanwhile, our study firstly put forward that the high expression of TGM2 tracked with an immunosuppression-promoting phenotype and TGM2 is involved in the modulation of PD-L1 expression by regulating downstream transcription factor STAT3/NF-κB in pancreatic cancer cells. In addition, the enrichment disparity of IMs in each subtype may suggest multiple potential combination immunotherapy strategies.

The prognostic impact of tumor immune features is emerging to be concerned. Among all subtypes in our study, patients comprising C2 have the worst overall survival. The reasons for the prognostic discrepancy can be attributed to the following points. Firstly, the immune signatures in C2 are dominated by TGF-β and Wound healing modules which were associated with an immunosuppressive and pro-tumor phenotype [21, 22], and inversely correlated with patients prognosis based on our data. Meanwhile, the lowest enrichment degree in Lymphocyte module is another feature of C2. Consistent with that, the infiltration of T-, B-lymphocytes, and NKT cells in C2 is less than the other subtypes, indicating a poor preexisting anti-tumor immunity [23]. Moreover, the highest enrichment degree of IFN-γ module is found in C2. IFN-γ is a major mediator inducing the death of tumor cells. However, continuous exposure to IFN-γ also stimulates tumor cells to express multiple inhibitory modulators including PD-L1 which are majorly enriched in C2 and C3 in our study, thereby suppress the secretion of IFN-γ by effector T cells and result in T cell exhaustion [24, 25]. Together, a more prominent feature of immunosuppression may be promoted by these features of C2 and therefore the prognosis of C2 is worse. In addition to these molecular immune features, our results show that the overall burden of NKT cells is associated with a better prognosis in PDAC, while that is rarely reported before. Similar to CD8^+^ T cell, NKT cell is also regarded as one of the front-line anti-tumor forces [26]. Apart from directly targeting the tumor cells with CD1d positive, recent literature on the mice model demonstrated that NKT cells could restrict the tumor evolution of PDAC indirectly by suppressing the pro-immunosuppression role of macrophages through prostaglandin E synthase-1 (mPGES-1) and 5-lipoxygenase (5-LOX) [27]. Consistent with that, subtype C2, with a high fraction of macrophage and a low fraction of NKT, shows a more prominent immunosuppression phenotype than C1 and C3. These distinct molecular and cellular features across three subtypes show a diverse immune response of PDAC, which would be the condition for prognostic evaluation and immunotherapy strategy designing.

To improve the clinical efficacy of anti-PD1/PDL1 therapy in PDAC, reasonable patient stratification and combination strategy designing should be concerned. For the former, prior studies indicated that the level of preexisting antitumor immunity and the expression level of PD-1/PD-L1 are two vital factors for the efficacy of anti-PD-1/PD-L1 treatment [28]. In our study, tumor samples comprising C3 are rich in various anti-tumor cytokines, such as TNF and IFN-γ, and have abundant infiltration of immune cells including NKT cells and T lymphocytes as well as B lymphocytes. Despite the anti-tumor effect of B cells remained unclear, a recent study in melanoma indicated that B cells may contribute to the response to ICB treatment by altering T cell activation and function [29]. As well, C3 has the highest PD-1 and PD-L1 enrichment across the three subtypes. Thus, patients within the C3 subtype seem to be more suitable for anti-PD treatment. Meanwhile, based on our data, C3 is also rich in CTLA4, TIGIT, and HAVCR2 expression which may provide alternative options for combined targets. Compared with single-agent ICB therapy, combination treatment of anti-CTLA4 (ipilimumab) and anti-PDL1 (nivolumab or pembrolizumab) led to better tumor response and patient survival in melanoma, sarcoma, and small cell lung cancer [30–34]. Besides, preclinical studies have demonstrated that anti-TIGIT or anti-HAVCR2 can effectively control tumor evolution, suggesting a promising combination target for anti-PD-1/PD-L1 treatment [35, 36].

In contrary with subtype C3, anti-PD-1/PD-L1 treatment may be not appropriate for the patients within subtype C1 due to the relatively low infiltration of T/B lymphocytes and the poorest enrichment in PD-1/PD-L1. Of note, C1 has the highest VTCN1 expression as well as favorable enrichment scores in NKT cells. As a newly discovered immune checkpoint expressed on APC cells and tumor cells, VTCN1 is expected to become a novel target for immunotherapy in the future despite its regulatory mechanism in cancer immunity remained to be further explored [37]. Due to the advantages of targeted on tumor cells and suppressive effect on graft versus host disease, NKT cell (majorly invariant NKT cell) is regarded as a viable vector for the CAR or rTCR treatments with multiple preclinical animal models supporting favorable anti-tumor effects in solid tumors [38–40].

Subtype C2 has favorable enrichment scores in PD-L1, CD276, and VTCN1 but is poor at lymphocytic infiltration. In addition to anti-PD-L1 treatment to restore the anti-tumor immunity, strategies to target the oncogenic pathway is needed for patients in C2 to restrict tumor progression. TGM2 is a promising target for improving the response to chemotherapy in solid tumors including PDAC [12]. While its role in the immune evade process of PDAC remains unclear. In this study, we find that TGM2 is positively related to the expression of multiple inhibitory immunomodulators, and the high-TGM2 group is mainly enriched in the immunosuppressive subtype C2, suggesting that TGM2 may be involved in the regulation of immunosuppression in PDAC. Through in vitro experiments, we verified that TGM2 has a positive impact on PD-L1 in PANC-1 and Mia PaCa-2 cells. The underlying mechanisms may refer to two aspects. Firstly, STAT3 has been reported to be involved in the regulation of PD-L1 expression as transcriptional factors and confirmed by Chip/EMSA assays [20, 41–43]. However, whether TGM2 alters the expression of PD-L1 in PDAC via STAT3 signaling remains to be unknown. Our present study reveals that down-regulating TGM2 in PANC-1 and Mia PaCa-2 cells results in a decreased phosphorylation of STAT3, which indicates a potential pathway for the regulation of PD-L1 by TGM2. Secondly, previous studies revealed that TGM2 promotes the activation of AKT by suppressing PTEN, then results in the activation of downstream substances including transcription factor NF-κB in PDAC [9, 10]. Consistent with that, knocking down TGM2 in PANC-1 cells leads to a decrease in p-Akt and p-P65 expression. As a vital transcriptional factor, NF-κB can regulate the transcription of PD-L1 by directly binding to the promoter region of PD-L1 [19, 44]. Thus, TGM2 may take a positive impact on PD-L1 expression via Akt/ NF-κB pathway. These findings provide insights into the regulation network of PD-L1 expression in PDAC (Fig. 7f). In addition, inhibition of TGM2 may alleviate stroma fibrosis to facilitate the infiltration of immune effector cells and the entrance of drugs, can also restrain the tumor growth as previous studies reported [16, 45]. Thereby, targeting on TGM2, through siRNA/shRNA, CRISPR/Cas9 genetic silence, or inhibitor (e.g., miR1285, GK921, NC9) [11, 46–48], may be a promising combination strategy to enhance the sensitivity to anti-PD-1/PD-L1 therapy.

Some limitations in this study need to be addressed. Firstly, as a retrospective study, the clinical value of these findings needs to be further validated in a larger prospective cohort. Secondly, the potential selection bias of tumor specimens may exist. Thirdly, though transcriptional profiles in our study provided us underlying features of the immune response in PDAC, with multi-omics data gathering, more solid evidence will promote our insight into tailored treatment for PDAC.

In summary, our study uncovered the specific immune features among PDAC patients. For the discrepant immune response mechanisms, more personalized ICB treatment should be considered. Moreover, TGM2 may regulate the expression of PD-L1 in PDAC via STAT3 and Akt/NF-κB signaling pathway and predicts poorer survival of PDAC patients, indicating a potential role in immunotherapy.

## Supplementary Information

**Additional file 1: Figure S1.** Intergroup prognostic analysis of three immune subtypes. (A) Comparison between C1 and C2 (p=0.00094). (B) Comparison between C2 and C3 (p=0.038). (C) Comparison between C1 and C3 (p=0.24).** Figure S2.** Prognostic analysis of B cells, CD8+ T cells, NK cells, mast cells, monocytes, DCs, eosinophils, basophils and neutrophils.** Figure S3.** DEGs and correlation analysis in GEO database (GSE28735 and GSE62452) led to gene TGM2.** Figure S4.** Prognostic analysis of TGM2 in TCGA-ICGC-GEO cohort (p=0.0012).** Figure S5.** Relation between TGM2 and tumor immunosuppression in PDAC.

**Additional file 2.** The sample details of immune subtypes.

## Data Availability

The public datasets of PAAD, PACA-AU, GSE28735 and GSE62452 for this study can be found in The Cancer Genome Atlas (https://portal.gdc.cancer.gov/), International Cancer Genome Consortium (https://dcc.icgc.org/) and Gene Expression Omnibus (https://www.ncbi.nlm.nih.gov/geo/).

## Abbreviations

AJCC: American Joint Committee on Cancer
GEO: Gene Expression Omnibus
ICB: Immune checkpoint blockade
ICGC: International Cancer Genome Consortium
PDAC: Pancreatic ductal adenocarcinoma
PD-1: Programmed cell death protein 1
PD-L1: Programmed death 1 ligand 1
TGM2: Transglutaminase 2
TCGA: The Cancer Genome Atlas
