# Supplementary material for: Immune subtyping for pancreatic cancer with implication in clinical outcomes and improving immunotherapy
Source: Cancer Cell Int. 2021 Feb 26;21:137. doi: 10.1186/s12935-021-01824-z (PMC7908647; doi:10.1186/s12935-021-01824-z)
Supplement: Supplementary file 1 — Additional file 1: Figure S1. Intergroup prognostic analysis of three immune subtypes. (A) Comparison between C1 and C2 (p=0.00094). (B) Comparison between C2 and C3 (p=0.038). (C) Comparison between C1 and C3 (p=0.24). Figure S2. Prognostic analysis of B cells, CD8+ T cells, NK cells, mast cells, monocytes, DCs, eosinophils, basophils and neutrophils. Figure S3. DEGs and correlation analysis in GEO database (GSE28735 and GSE62452) led to gene TGM2. Figure S4. Prognostic analysis of TGM2 in TCGA-ICGC-GEO cohort (p=0.0012). Figure S5. Relation between TGM2 and tumor immunosuppression in PDAC. [file 12935_2021_1824_MOESM1_ESM.docx]

**
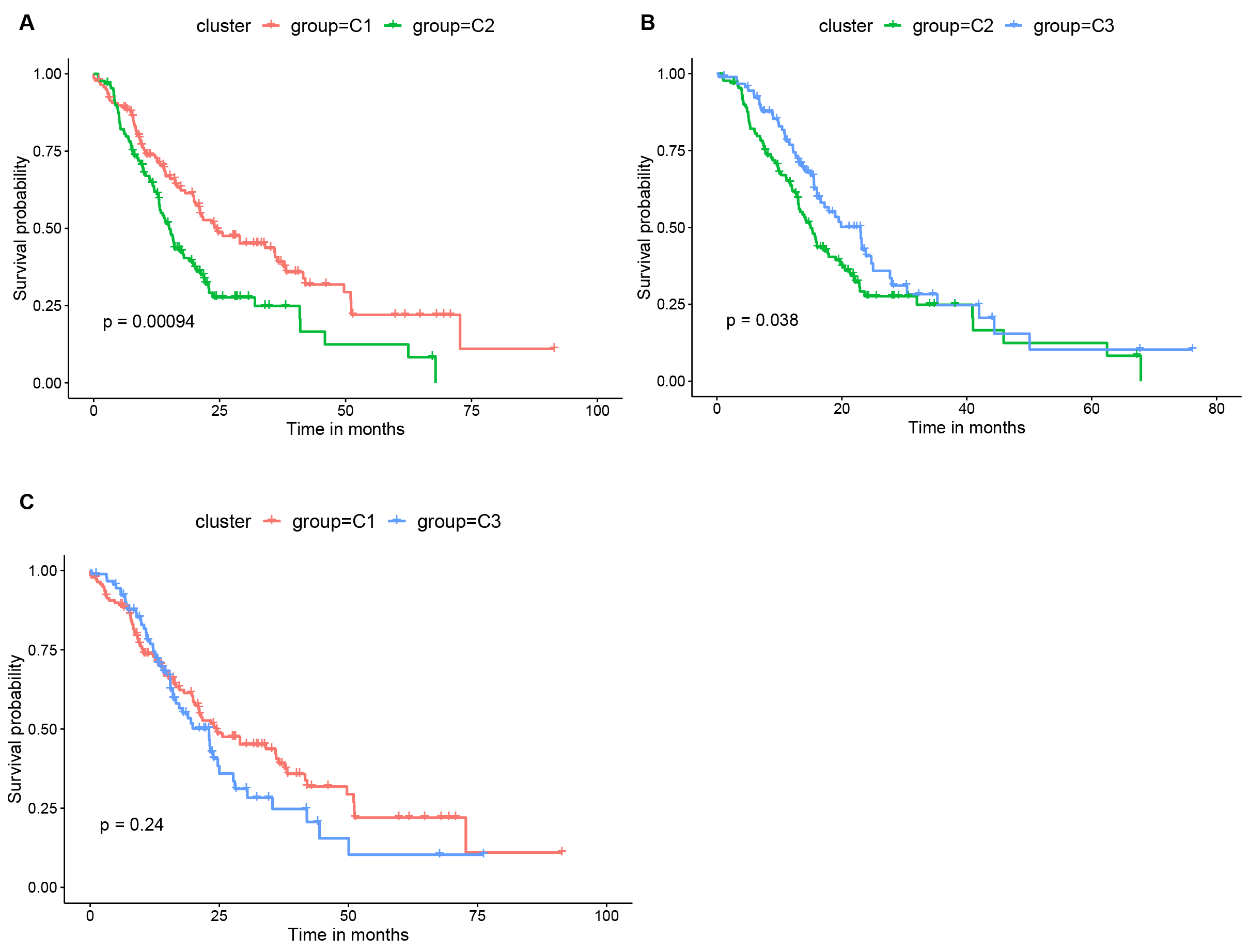
**

**Figure S1.** Intergroup prognostic analysis of three immune subtypes. **(A)** Comparison between C1 and C2 (*p*=0.00094). **(B)** Comparison between C2 and C3 (*p*=0.038). **(C)** Comparison between C1 and C3 (*p*=0.24).


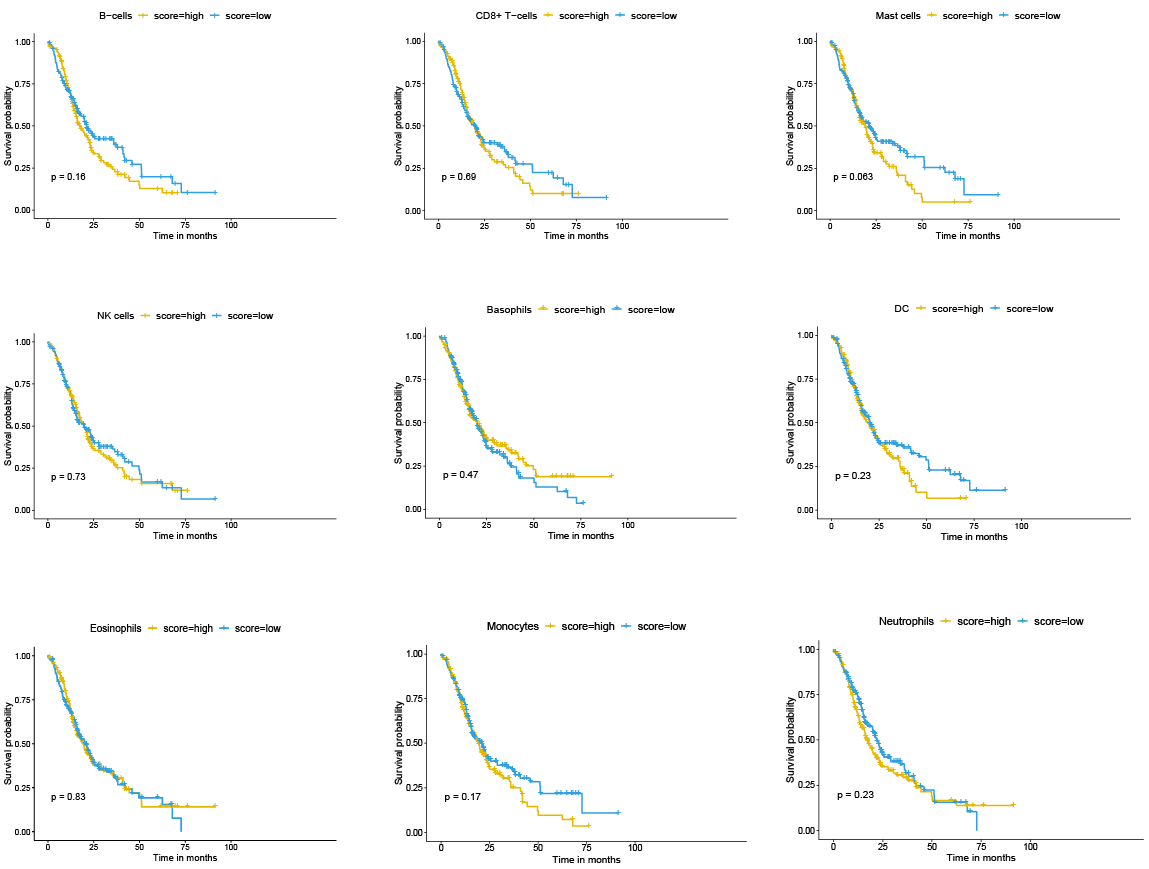


**Figure S2.** Prognostic analysis of B cells, CD8^+^ T cells, NK cells, mast cells, monocytes, DCs, eosinophils, basophils and neutrophils.


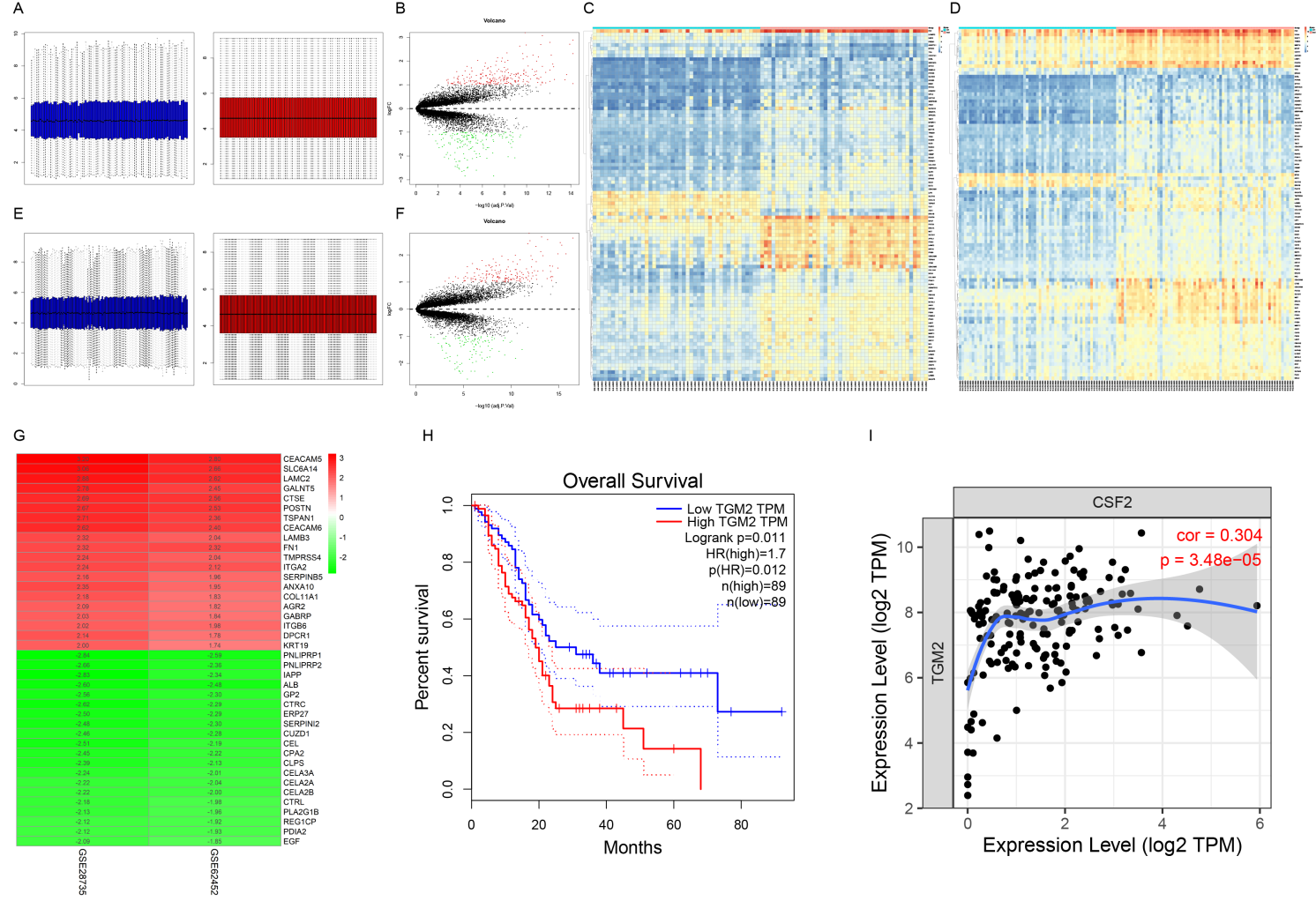


**Figure S3.** DEGs and correlation analysis in GEO database (GSE28735 and GSE62452) led to gene TGM2. **(A)** Normalization of gene profiles in GSE28735. **(B)** DEGs in GSE28735. **(C)** Cluster heat map of the top 100 DEGs in GSE28735. **(D)** Cluster heat map of the top 100 DEGs in GSE62452. **(E)** Normalization of gene profiles in GSE62452. **(F)** DEGs in GSE62452. **(G)** Heat map of partial DEGs in GSE28735 and GSE62452. **(H)** Prognostic analysis of TGM2 by GEPIA online tool (<http://gepia.cancer-pku.cn/>). **(I)** Correlation analysis of TGM2 and GM-CSF (gene: CSF2) by TIMER online tool (<https://cistrome.shinyapps.io/timer/>).


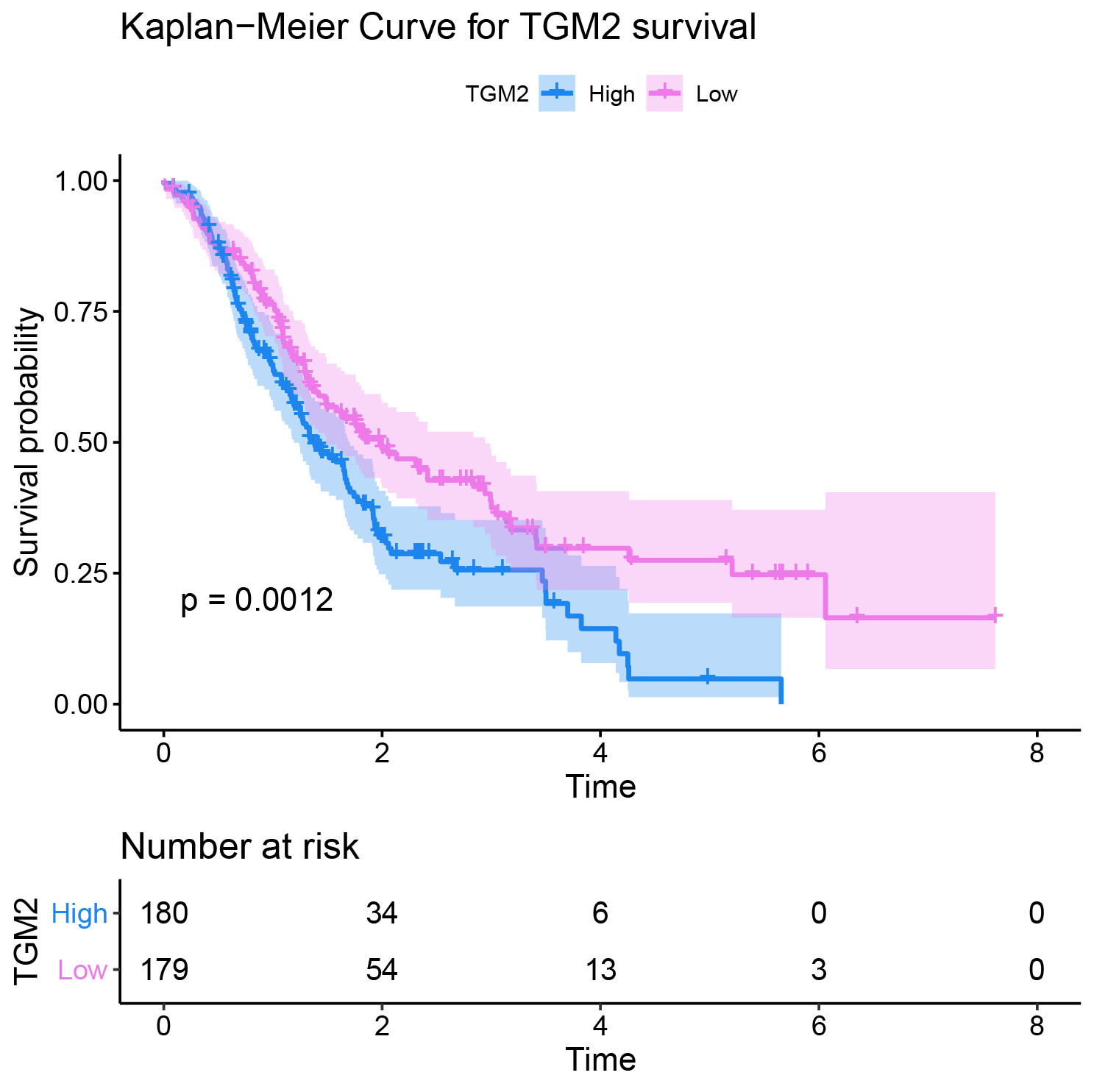


**Figure S4.** Prognostic analysis of TGM2 in TCGA-ICGC-GEO cohort (*p*=0.0012).


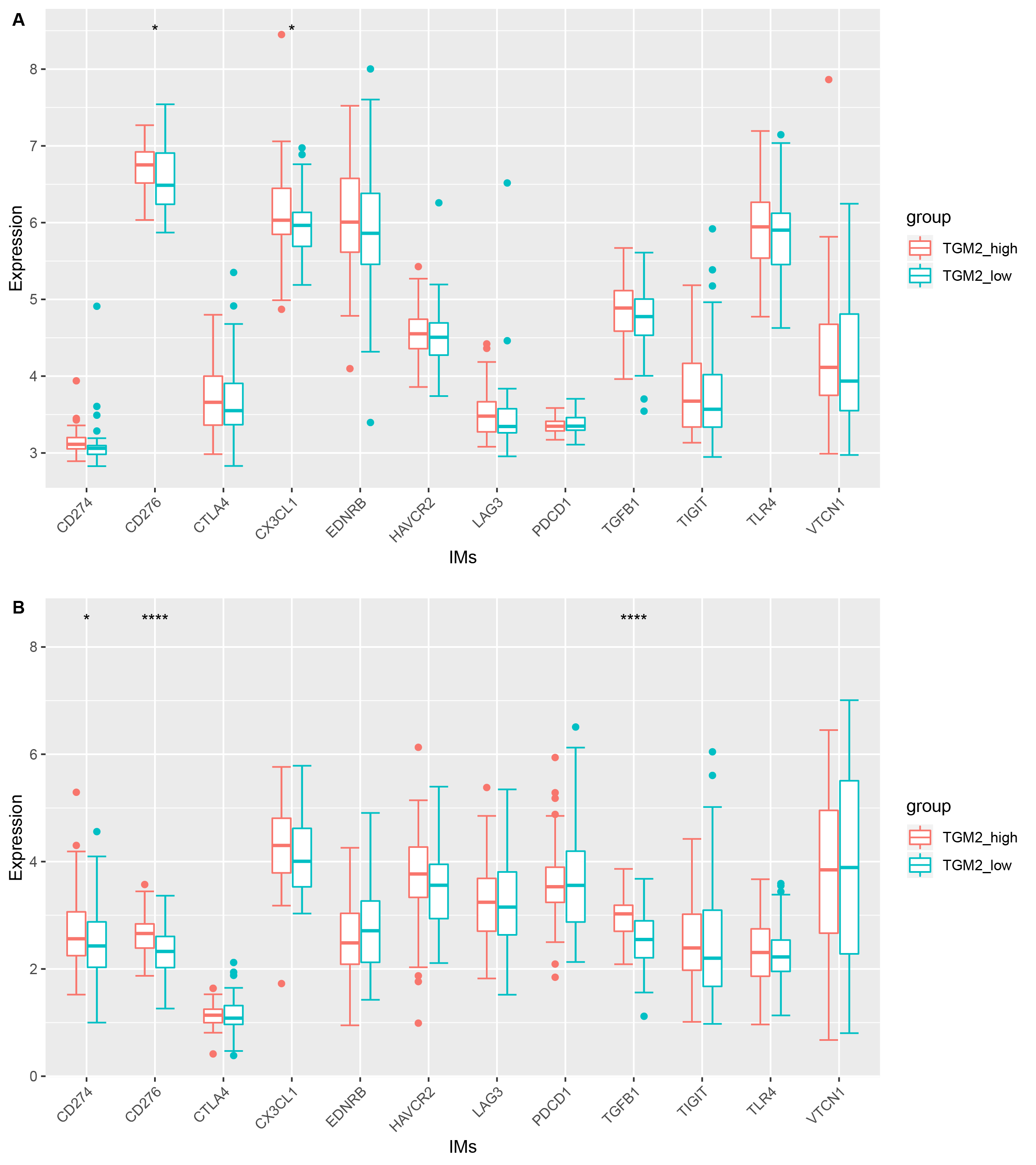


**Figure S5.** Relation between TGM2 and tumor immunosuppression in PDAC. **(A)** Validated in GSE62165 dataset which consists of 118 tumor samples. **(B)** Validated in GSE71729 dataset which consists of 146 tumor samples.
